# Effect of preoperative tranexamic acid on hidden blood loss and C-reactive protein in patients undergoing Delta-channel endoscopic lumbar discectomy: a retrospective case-control study

**DOI:** 10.3389/fsurg.2026.1765009

**Published:** 2026-07-14

**Authors:** Shenshen Hao, Shengli Dong, Hongke Li, Yu Liang, Shuaizhi Li, Xinpeng Li, Xiaoya Zhu, Xinhao Cao, Fei Chen, Bing Qian, Shuai Liu

**Affiliations:** 1 Department of Spine and Bone Oncology, General Hospital of Pingmei Shenma Medical Group, Pingdingshan City, Henan, China; 2 Department of Spine, Affiliated Hospital of Qingdao University, Qingdao City, Shandong, China; 3 Department of Radiology, General Hospital of Pingmei Shenma Medical Group, Pingdingshan City, Henan, China; 4 Department of Bone Disease Area of Limbs, General Hospital of Pingmei Shenma Medical Group, Pingdingshan City, Henan, China; 5 Department of Anesthesiology and Perioperative Medicine, General Hospital of Pingmei Shenma Medical Group, Pingdingshan City, Henan, China; 6 Interventional Operating Room, The Second People's Hospital of Pingdingshan, Pingdingshan City, Henan, China; 7 Emergency Medicine Department, Honghui Hospital, Xi'an Jiaotong University, Xi'an City, Shaanxi, China

**Keywords:** Delta-channel interlaminar endoscopic discectomy, lumbar disc herniation, tranexamic acid, hidden blood loss, C-reactive protein

## Abstract

**Background:**

The minimally invasive Delta-channel interlaminar endoscopic discectomy has been increasingly applied in clinical practice. However, the impact of tranexamic acid (TXA) on hidden blood loss (HBL) and C-reactive protein (CRP) levels in patients undergoing this procedure remains unclear. Accordingly, the present study is designed to investigate the impact of intravenous TXA on postoperative HBL and CRP levels in patients with lumbar disc herniation (LDH) treated via Delta-channel interlaminar endoscopic discectomy.

**Methods:**

A retrospective analysis was conducted on clinical data of 69 LDH patients who underwent Delta-channel interlaminar endoscopic discectomy between January 2023 and December 2024. Patients were divided into two groups. Of these, 25 patients who received intravenous infusion of 1 g TXA 15 min prior to skin incision were assigned to the observation group, whereas 44 patients who did not receive TXA were allocated to the control group. The following parameters were recorded as observation indicators, including operation time, intraoperative blood loss (IBL), HBL, postoperative activated partial thromboplastin time (APTT), prothrombin time (PT), thrombin time (TT), fibrinogen (FIB), platelet count (PLT), D-dimer, CRP, hemoglobin (HB), red blood cell count (RBC), hematocrit (HCT), incision healing status, and the incidence of lower extremity deep vein thrombosis (DVT).

**Results:**

All surgeries were completed successfully, with all incisions achieving primary healing and no DVT events reported in either group. The HBL in the observation group was significantly lower than that in the control group, with a statistically significant difference (*p* < 0.05). There was no statistically significant difference between the two groups in terms of operation time, IBL, CRP, RBC, HB, HCT, APTT, PT, TT, FIB, PLT and D-dimer (*p* > 0.05). Univariate analysis revealed that TXA, TT, RBC, HB, and HCT were influencing factors for HBL. Further multivariate analysis identified TXA, TT, and HCT as independent predictors of HBL.

**Conclusion:**

When treating LDH with Delta-channel interlaminar endoscopic discectomy, preoperative intravenous administration of TXA can effectively reduce HBL and serves as a protective factor against HBL. Meanwhile, it does not significantly affect postoperative CRP levels, indicating that it does not interfere with the clinical evaluation of postoperative inflammatory responses.

## Introduction

With the advancement of medical technology, minimally invasive spinal endoscopic surgery has emerged as a standard modality for the management of lumbar disc herniation (LDH) (1, 2). Traditional percutaneous transforaminal endoscopic techniques, constrained by a single operating channel, are associated with limitations in surgical field of view and instrument maneuverability, as well as suboptimal surgical efficiency (3). Additionally, the frequent use of local anesthesia during these procedures often results in poor patient experience. While the unilateral biportal endoscopic technique has shifted to general anesthesia, it still exhibits drawbacks such as greater surgical trauma, higher total blood loss (TBL), and increased hidden blood loss (HBL) compared to single-channel endoscopic approaches (4). As a modified interlaminar approach, the Delta-channel interlaminar endoscopic discectomy (abbreviated as Delta technique) significantly expands the operating space and enhances visual clarity by enlarging the working channel. It not only preserves the core advantages of minimally invasive surgery, including minimal trauma and rapid recovery, but also improves patient experience via the application of general anesthesia (5–7). Consequently, it holds substantial promise in the field of minimally invasive spinal surgery (8, 9).

However, during the application of the Delta technique, bleeding remains likely when manipulating the spinal canal and epidural space, with even the “red screen” phenomenon occasionally observed (8). Coupled with the limited operating space, this not only elevates the difficulty of hemostasis but also may increase the risk of nerve injury sometimes (10). Studies have demonstrated that although intraoperative blood loss (IBL) in endoscopic surgery is minimal, HBL is often substantial, which impairs postoperative recovery (11). Thus, effective control of IBL and HBL is clinically significant. Tranexamic acid (TXA), an antifibrinolytic agent, exerts hemostatic effects by inhibiting plasmin activation (12). Among its administration regimens, the single preoperative intravenous dose administered 15 min before surgery, recommended in the 2019 expert consensus (13), has emerged as one of the most commonly used approaches due to its operational simplicity and rapid onset of action (14). However, the efficacy of TXA in the context of the Delta technique has not been sufficiently documented. Additionally, antibiotics are typically not administered perioperatively in such surgeries, and C-reactive protein (CRP) serves as a key biomarker for monitoring postoperative inflammatory responses. Whether TXA administration impacts CRP levels bears implications for the clinical identification of postoperative infections.

Given that HBL in endoscopic lumbar procedures often exceeds visible loss by several-fold and CRP guides infection surveillance in non-antibiotic settings, we hypothesized that single-dose preoperative TXA would reduce HBL without altering CRP. This retrospective case-control study aimed to evaluate these effects in patients undergoing Delta technique for LDH.

## Materials and methods

### Study design

This was a retrospective single-center case-control study. Clinical data of patients admitted between January 2023 and December 2024 were retrospectively collected. The inclusion criteria were as follows. *a.* Preoperative diagnosis of LDH was confirmed. *b.* The surgical procedure was performed using the Delta technique. *c.* The surgical intervention involved a single spinal segment. *d.* The patient was classified as Grade 2 according to the American Society of Anesthesiologists classification. *e.* The case data was complete. The Exclusion criteria were as follows. *a.* The patient had preoperatively concomitant hematological disorders. *b.* The patient had received anticoagulant medications within one week prior to surgery. *c.* The patient had preexisting comorbidities, including lumbar vertebral fractures, tuberculosis, or tumors, prior to surgery. *d.* The patient had preexisting deep vein thrombosis (DVT) of the lower extremities prior to surgery. *e.* The patient developed cerebrospinal fluid leakage either intraoperatively or postoperatively. *f.* The planned surgical segment had a prior surgical history. *g.* The patient had preexisting renal dysfunction prior to surgery. A total of 69 cases that met the above criteria were included., including 37 males and 32 females, aged 17–78 years with a mean age of (48.190 ± 12.295) years.

Patients were stratified into an observation group (*n* = 25) and a control group (*n* = 44) according to whether they received intravenous TXA. All patients underwent general anesthesia in the prone position. For the observation group, a single intravenous infusion of 1 g TXA was administered 15 min before skin incision following induction of general anesthesia, while the control group did not receive TXA. Both groups were treated with the identical surgical technique, namely the standard interlaminar approach using Delta technique (15). Intraoperatively, no drainage tubes were placed. Postoperatively, both groups received consistent routine management, including glucocorticoid administration for stress response alleviation, dehydration and detumescence therapy, thromboprophylaxis, pharmacologic analgesia, as well as monitoring of incision healing and lower extremity DVT. On the first postoperative day, hematological tests were performed to evaluate HBL and CRP levels. Under physician supervision, patients performed ankle pump exercises, lower extremity elevation exercises, and ambulatory functional training with a lumbar corset or external brace fixation. Additionally, infrared irradiation therapy was adjunctively applied to facilitate surgical incision healing.

### Outcome measures

Preoperative baseline data were collected for both groups, including age, gender, body mass index (BMI), surgical segments, comorbid diabetes mellitus, comorbid hypertension, activated partial thromboplastin time (APTT), prothrombin time (PT), thrombin time (TT), fibrinogen (FIB), platelet count (PLT), hemoglobin (HB), red blood cell count (RBC), hematocrit (HCT), white blood cell count (WBC), erythrocyte sedimentation rate (ESR), and CRP. Surgical-related observation variables were recorded as follows. They included operation time, IBL, HBL, postoperative APTT, PT, TT, FIB, PLT, D-dimer, CRP, HB, RBC, HCT, incision healing status, and lower extremity DVT occurrence.

HBL was calculated using methods described in prior literature, specifically the Gross formula (16) and Nadel formula (17). The detailed calculations were as follows. HBL = TBL−IBL. TBL = Patient's blood volume (PBV) × (Preoperative HCT−Postoperative HCT)/Mean HCT. Mean HCT = (Preoperative HCT + Postoperative HCT)/2. PBV (L) = k₁ × Height (m)^3^ + k₂ × Weight (kg) + k₃ (for males: k₁ = 0.366, k₂ = 0.03219, k₃ = 0.6041; for females: k₁ = 0.3561, k₂ = 0.03308, k₃ = 0.1833). Given the large volume of intraoperative irrigation fluid, IBL was quantified via the HB concentration assay (18, 19). IBL = (HB concentration in irrigation fluid sample (g/L) × Total volume of irrigation fluid (mL))/Preoperative HB concentration (g/L).

### Statistical methods

Data analyses were performed using SPSS 22.0 software (IBM Corp., Armonk, NY, USA). Normality testing was first conducted for continuous variables. Normally distributed data were presented as mean ± standard deviation and compared between groups using independent-samples t-tests. Non-normally distributed data were expressed as median [interquartile range, IQR] (M [P25; P75]) and analyzed via Mann–Whitney U tests. Categorical data were compared using the chi-square test. Variables with statistically significant differences were designated as dependent variables, while baseline characteristics and intraoperative parameters served as independent variables for linear regression analyses. Variables were entered into the model sequentially (enter method). Multicategorical independent variables were converted into dummy variables, while continuous or binary independent variables were included directly in the linear regression model. A *P*-value < 0.05 was considered statistically significant.

## Results

There were no statistically significant differences in the preoperative baseline data between the two groups (*p* > 0.05). The detailed results were shown in Table 1.

**Table 1:** Comparison of baseline characteristics between the two groups.

Variable	Observation Group (*n* = 25)	Control Group (*n* = 44)	t/*χ*^2^/Z	*P*
Age, years	49.920 ± 10.484	47.200 ± 13.227	0.880	0.382
Gender, *n*			0.089	0.765
Male	14	23		
Female	11	21		
BMI, kg/m^2^	25.110 ± 2.592	24.230 ± 2.929	1.248	0.217
Surgical segment, *n*			0.326	0.850
L3/4	1	2		
L4/5	12	18		
L5/S1	12	24		
Comorbid diabetes mellitus, *n*	2	3	0.033	0.856
Comorbid hypertension, *n*	6	6	1.192	0.275
APTT, s	30.252 ± 2.351	31.123 ± 3.067	−1.228	0.224
PT, s	10.8 [10.6; 11.5]	10.8 [10.5; 11.6]	−0.381	0.703
TT, s	13.80 [13.30; 14.07]	14.08 [13.36; 14.50]	−1.836	0.066
FIB, g/L	2.956 ± 0.503	3.045 ± 0.513	−0.698	0.485
PLT, 10^9^/L	250.000 ± 61.347	240.360 ± 69.406	0.637	0.526
HB, g/L	137.360 ± 12.563	140.320 ± 16.173	−0.788	0.433
RBC, 10^12^/L	4.513 ± 0.448	4.622 ± 0.510	−0.885	0.379
HCT, L/L	0.428 ± 0.037	0.426 ± 0.047	0.208	0.836
WBC, 10^9^/L	7.055 ± 1.681	6.999 ± 2.229	0.107	0.915
CRP, mg/L	0.84[0.42; 2.80]	1.08 [0.59; 2.96]	−1.005	0.315
ESR, mm/h	10[5; 21]	14 [7; 18]	−1.169	0.242

All surgeries were successfully completed in both groups, with well-healed postoperative incisions and no DVT observed. The HBL in the observation group was significantly lower than that in the control group (*P* < 0.05). There were no statistically significant differences in the operation time, IBL, postoperative CRP, RBC, HB, HCT, APTT, PT, TT, FIB, PLT and D-dimer between the two groups (*p* > 0.05). They were shown in Table 2.

**Table 2:** Comparison of outcome measures between the two groups.

Variable	Observation Group (*n* = 25)	Control Group (*n* = 44)	t/χ^2^/Z	*P*
Operation time, min	111.960 ± 28.289	103.180 ± 26.427	1.269	0.211
IBL, mL	30[20; 50]	50[20; 55]	−0.721	0.471
HBL, mL	217[145; 311]	298[230; 412]	−2.322	0.020
APTT, s	28.856 ± 2.027	29.805 ± 2.407	−1.662	0.101
PT, s	11.552 ± 0.692	11.776 ± 0.926	−1.053	0.296
TT, s	14.217 ± 1.268	14.426 ± 0.963	−0.773	0.442
FIB, g/L	2.83[2.66; 2.94]	2.93[2.61; 3.20]	−0.868	0.385
PLT, 10^9^/L	242.160 ± 64.435	232.320 ± 64.856	0.608	0.546
D-dimer, mg/L	0.07[0.05; 0.10]	0.09[0.06; 0.16]	−1.716	0.086
HB, g/L	132.960 ± 11.717	130.230 ± 14.896	0.788	0.433
RBC, 10^12^/L	4.337 ± 0.369	4.263 ± 0.480	0.661	0.511
HCT, L/L	0.402 ± 0.035	0.391 ± 0.042	1.095	0.277
CRP, mg/L	2.90[1.22;5.23]	3.11[0.87; 6.71]	−0.106	0.915

Therefore, in this study, HBL was designated as the dependent variable, while baseline characteristics and intraoperative parameters were included as independent variables for univariate linear regression analysis. The results of univariate analysis showed that TXA, TT, RBC, HB, and HCT were significantly correlated with HBL (*p* < 0.05). While age, gender, BMI, comorbid hypertension, comorbid diabetes mellitus, surgical segments, APTT, PT, FIB, PLT, WBC, CRP, ESR, operation time, and IBL were not significantly correlated with HBL (*p* > 0.05). The specific details were presented in Table 3.

**Table 3:** Univariate linear regression analysis with HBL as the dependent variable.

Independent Variable	B	SE	t	*p*
Age, years	1.616	1.544	1.046	0.299
Gender (1 = male, 0 = female)	43.621	37.728	1.156	0.252
BMI, kg/m^2^	3.994	6.759	0.591	0.557
Comorbid diabetes mellitus (1 = yes, 0 = no)	131.135	71.519	1.834	0.071
Comorbid hypertension (1 = yes, 0 = no)	7.044	50.123	0.141	0.889
APTT, s	12.504	6.559	1.906	0.061
PT, s	−25.142	23.053	−1.091	0.279
TT, s	46.193	24.176	2.035	0.046
FIB, g/L	48.171	37.131	1.297	0.199
PLT, 10^9^/L	−0.346	0.285	−1.211	0.230
HB, g/L	3.563	1.205	2.957	0.004
RBC, 10^12^/L	101.208	37.220	2.719	0.008
HCT, L/L	1,378.549	410.644	3.357	0.001
WBC, 10^9^/L	8.661	9.102	0.952	0.345
CRP, mg/L	−0.098	2.205	−0.044	0.965
ESR, mm/h	2.090	1.620	1.290	0.202
TXA (1 = yes, 0 = no)	−90.446	37.955	−2.383	0.020
Surgical segment (reference: L3/4)				
L4/5	14.269	96.068	0.149	0.882
L5/S1	−7.609	95.338	−0.080	0.937
Operation time, min	0.257	0.702	0.367	0.715
IBL, mL	1.371	0.698	1.963	0.054

TXA, TT, RBC, HB, and HCT, variables that exhibited significant correlations in the univariate analysis, were included in a multivariate linear regression analysis. Model fitting assessment yielded an F-statistic of 4.831 (*P* < 0.001), indicating the model was statistically significant. The residual normality test returned a *P*-value of 0.158, confirming that residuals followed a normal distribution. Collinearity diagnostics showed a variance inflation factor (VIF) of 6.080, suggesting no substantial collinearity issues. The results of the multivariate linear regression analysis indicated that TXA, TT, and HCT were significantly correlated with HBL (*p* < 0.05), while RBC and HB were not significantly correlated with HBL (*p* > 0.05). The results were presented in Table 4.

**Table 4:** Multivariate linear regression analysis with HBL as the dependent variable.

Independent Variable	B	SE	t	*p*	95% (CI)		VIF
					Lower Bound	Upper Bound	
Constant	−886.382	357.402	−2.480	0.016	−1,600.593	−172.171	
TXA (1 = yes, 0 = no)	−88.055	35.963	−2.449	0.017	−159.920	−16.189	1.077
RBC, 10^12^/L	−17.521	76.167	−0.230	0.819	−169.728	134.686	4.906
HB, g/L	−0.307	2.770	−0.111	0.912	−5.842	5.228	6.080
HCT, L/L	1,676.901	762.864	2.198	0.032	152.438	3,201.363	3.843
TT, s	44.876	22.070	2.033	0.046	.773	88.980	1.021

## Discussion

The stress response triggered by surgical trauma and short-term postoperative immobilization are well-recognized contributors to hypercoagulable states and elevated risk of DVT. TXA, an antifibrinolytic agent, exerts its pharmacologic effect by competitively inhibiting plasminogen activation. Theoretically, this reduction in fibrinolytic activity may disrupt the dynamic balance of thrombosis and fibrinolysis, thereby potentially increasing the risk of DVT (20, 21). The concern that has long persisted regarding its clinical safety. The results of this study show that all patients completed the surgery successfully, and no DVT occurred postoperatively. Additionally, there were no significant differences in the levels of APTT, PT, TT, FIB, PLT, which reflect coagulation function, and D-dimer, which reflects the state of thrombosis formation and dissolution, between the two groups. These results provide supportive evidence for the safety of TXA in Delta technique, indicating that single-dose intravenous TXA administration did not induce clinically meaningful perturbations to the coagulation-fibrinolysis system nor translate to a higher incidence of DVT. Several potential mechanisms may underlie this observation. Firstly, the overall incidence of DVT following spinal surgery is inherently low, with literature reporting rates of approximately 2%–4% (22). Secondly, all patients in this study received standardized perioperative DVT prophylaxis, including early postoperative active ankle pump exercises, in-bed limb mobilization, and physician-supervised early ambulation. This multimodal, proactive rehabilitation strategy may have more effectively promoted blood circulation and mitigated stasis risk compared to monotherapy (e.g., standalone pharmacologic or mechanical prophylaxis), thereby contributing to the low DVT incidence observed (10). Collectively, these data suggest that TXA exhibits a favorable safety profile when administered within a comprehensive perioperative management framework.

Theoretically, TXA is postulated to improve surgical visualization by reducing intraoperative bleeding, thereby streamlining hemostatic maneuvers and enhancing surgical efficiency. These effects that might ultimately translate to reduced IBL and shorter operative times. However, no statistically significant differences in IBL or operative time were observed between the two groups in the present study. This seemingly paradoxical finding may be attributed to the following synergistic factors. Firstly, the Delta technique is inherently minimally invasive, characterized by blunt muscle dissection and limited bony structure manipulation. These features inherently maintain baseline IBL at a relatively low level. While TXA can further attenuate microvascular oozing, its incremental hemostatic effect may not have reached the threshold required to yield a statistically significant reduction in overall IBL. Secondly, this study was performed by an experienced spinal endoscopy team with proficient surgical technique and standardized procedural workflows. Such expertise minimized bleeding-induced visual obscuration and operative disruptions, thereby blunting the potential marginal benefits of TXA in enhancing procedural flow and reducing operative time. Thirdly, as a retrospective study with a relatively limited sample size, the analysis may have been underpowered to detect subtle differences, compounded by the potential presence of unaccounted confounding variables.

In the field of minimally invasive spinal surgery, HBL has emerged as a growing focus of clinical attention. Accumulating evidence indicates that HBL constitutes the predominant component of TBL in both unilateral biportal endoscopy and percutaneous transforaminal endoscopic techniques, often exceeding IBL by several-fold or even tens of-fold (4, 23, 24). The findings of the present study align with this established consensus and further confirm that TXA significantly reduces HBL in Delta technique.

HBL primarily arises from interstitial tissue bleeding and erythrocyte hemolysis. Previous research has demonstrated that approximately 60% of HBL in minimally invasive spinal surgery stems from tissue fluid extravasation, with the remaining 40% attributed to hemolysis (25). Beyond these universal mechanisms, the Delta technique may be associated with unique anatomical and procedural factors contributing to HBL. Firstly, intraoperative resection of partial lamina and facet joint bone to expose the surgical field creates a non-closable osseous cavity, providing an anatomical niche for blood retention. Secondly, continuous intraoperative saline irrigation and the resultant hydrostatic pressure may impede definitive hemostasis at microvascular bleeding sites on bony surfaces (26). Thirdly, the potential space formed after removal of the muscle-dissected working channel may serve as a persistent source of oozing. Fourthly, high-frequency radiofrequency electrocoagulation, used for hemostasis, generates oxidative species that induce erythrocyte destruction and exacerbate hemolysis (27). TXA targets the critical pathophysiological link of persistent tissue bleeding by stabilizing formed fibrin clots and effectively inhibiting hyperfibrinolysis. Notably, hyperfibrinolysis is estimated to account for up to 60% of blood loss in orthopedic procedures (28), providing theoretical rationale for TXA to reduce HBL. Multivariate regression analysis in this study identified TXA administration as an independent factor associated with reduced HBL, further validating its hemostatic efficacy.

Additionally, an independent association between HCT and HBL was observed, likely reflecting the causal relationship inherent in HBL estimation, given that HBL is calculated based on HCT values. The independent correlation between TT and HBL may be explained by surgical stress-induced dysregulation of the coagulation, fibrinolytic, and immune systems, triggering a cascade effect that promotes a hypercoagulable state and subsequent alterations in TT (29). As a non-visible form of blood loss influenced by multiple factors, HBL may be partially modulated by TT. Notably, despite the theoretical expectation that HBL reduction would mitigate postoperative anemia, no statistically significant differences in postoperative RBC, HB, or HCT levels were detected between groups. This may be attributed to the absence of preoperative anemia, preserved bone marrow hematopoietic function, and TBL remaining within the compensatory capacity of the patients' physiological systems, thus precluding measurable differences in anemia-related indices postoperatively.

For clean surgical procedures such as spinal endoscopic surgery, where perioperative prophylactic antibiotics are not routinely administered, monitoring of postoperative inflammatory responses is of critical clinical significance. CRP, characterized by high sensitivity, favorable specificity, and a relatively stable half-life, has emerged as a widely used serological biomarker for evaluating postoperative inflammatory status and early detection of potential infections (30, 31). In recent years, beyond its well-established hemostatic efficacy, the potential anti-inflammatory properties of TXA have garnered increasing research attention (32). While the underlying anti-inflammatory mechanisms of TXA remain incompletely elucidated, proposed pathways include indirect inhibition of inflammatory responses via suppression of plasminogen activation (33) or attenuation of inflammatory cytokine production (34).

However, the data from this study revealed no statistically significant difference in postoperative CRP levels between the two groups. This finding indicates that under the single-dose (1 g) intravenous administration regimen employed in this study, TXA did not exert a clinically meaningful interference with the body's physiological inflammatory stress response to surgical trauma. This preserves the clinical diagnostic utility of CRP as a biomarker for infection monitoring. Indirectly, this result also suggests that its effect in reducing blood loss was not achieved at the cost of disrupting the inflammatory response trajectory. This aligns with our team's prior findings from a similarly designed study of TXA in posterior lumbar interbody fusion surgery (35). Notably, emerging evidence suggests that the anti-inflammatory efficacy of TXA may be dose- and frequency-dependent (36). The dose utilized in the present study may not have reached the threshold required to elicit a detectable anti-inflammatory effect. Thus, the precise dose-response relationship and mechanistic underpinnings warrant further investigation in future studies.

### Limitation

This study has several limitations that warrant consideration when interpreting the conclusions. Firstly, as a single-center retrospective study, it is susceptible to selection bias (TXA use by surgeon preference) and limited statistical power for rare events due to the small sample size. Secondly, the relatively small sample size and absence of propensity score matching mean that while multivariate analyses were performed to adjust for potential confounders, residual or unmeasured confounding cannot be entirely excluded. Thirdly, IBL was estimated using a formula rather than measured directly. Furthermore, the low absolute IBL means that trace amounts of blood absorbed by surgical dressings are challenging to quantify precisely. Despite adherence to a standardized estimation protocol to optimize accuracy, inherent measurement errors remain unavoidable. This, in turn, may introduce bias into the calculation of HBL via the applied formula. Fourthly, HBL calculations rely on perioperative HCT measurements. However, perioperative fluid administration induces hemodilution, which may introduce inherent bias into the HCT-based estimation model (37). Fifthly, this study focused on the application effect of TXA in the Delta technique and did not compare it with unilateral biportal endoscopic technique. These limitations restrict the general applicability of the study results, and future multicenter randomized controlled trials are needed.

## Conclusion

This retrospective analysis suggests that a single preoperative intravenous dose of 1 g TXA may contribute to the reduction of HBL in the treatment of single-segment LDH using the Delta technique, with no interference observed in the assessment of postoperative inflammatory status based on CRP. Concurrently, no elevated risk of DVT or perturbations in coagulation function was detected. Owing to the constraints of the retrospective design and limited sample size, the conclusions of this study warrant further validation through large-scale, prospective randomized controlled trials.

## Data Availability

The original contributions presented in the study are included in the article/Supplementary Material, further inquiries can be directed to the corresponding author.
